# Harvester Ant Colony Variation in Foraging Activity and Response to Humidity

**DOI:** 10.1371/journal.pone.0063363

**Published:** 2013-05-23

**Authors:** Deborah M. Gordon, Katherine N. Dektar, Noa Pinter-Wollman

**Affiliations:** Department of Biology, Stanford University, Stanford, California, United States of America; Field Museum of Natural History, United States of America

## Abstract

Collective behavior is produced by interactions among individuals. Differences among groups in individual response to interactions can lead to ecologically important variation among groups in collective behavior. Here we examine variation among colonies in the foraging behavior of the harvester ant, *Pogonomyrmex barbatus*. Previous work shows how colonies regulate foraging in response to food availability and desiccation costs: the rate at which outgoing foragers leave the nest depends on the rate at which foragers return with food. To examine how colonies vary in response to humidity and in foraging rate, we performed field experiments that manipulated forager return rate in 94 trials with 17 colonies over 3 years. We found that the effect of returning foragers on the rate of outgoing foragers increases with humidity. There are consistent differences among colonies in foraging activity that persist from year to year.

## Introduction

Collective behavior arises from the local interactions of individuals. Bird flocks and fish schools turn, termites build nests, and wildebeest cross the plains, because individuals respond to what others are doing nearby. The first question about collective behavior is how the actions of individuals add up to the dynamic behavior we observe. The recent explosion of work on collective behavior in many different animal groups (e.g. [1]–[5]) seeks to describe how local interactions among individuals produce a certain collective outcome.

Evolutionary questions about behavior require us to think about variation, and there is growing interest in individual variation in behavior [6]. Consistent differences among individuals in behavior [7], recently called personality [8]–[9], temperament [10] or behavioral syndromes [11], may be heritable reaction norms like those created by phenotypic plasticity in any other trait [12].

Recent work shows variation among social insect colonies in behavior. Honeybee colonies differ in pollen and nectar collection [13] and in foraging behavior [14]. Ant colonies differ in foraging activity [15]–[18], and in the aggression levels of colonies [19] and individuals [20]–[21]. Colonies function as reproductive individuals; the colony produces reproductives, who mate with the reproductives of other colonies and then form offspring colonies. When the collective behavior of a colony is heritable and ecologically important, selection may act on variation among colonies in collective behavior.

Here we examine differences among colonies of the red harvester ant, *Pogonomyrmex barbatus*, in the collective behavior that regulates foraging in response to food availability. An inactive forager is stimulated to leave the nest on its next trip by the return of foragers with food [17], [22]–[24]. Interactions between returning and outgoing foragers consist of brief antennal contacts inside the nest entrance as the ants come in and out. During an antennal contact a forager detects the task-specific cuticular hydrocarbon profile of the other [25] and whether it is carrying food [26]. Forager return rate reflects food availability because the duration of a foraging trip depends on search time [27]. The more food is available, the less time is needed to search and the more quickly a forager returns with food. Thus the overall rate of return of successful foragers reflects the availability of food on that day.

Harvester ants foraging in hot, dry conditions lose water, but obtain water from metabolizing fats in the seeds that they eat [28]–[29]. Positive feedback on foraging activity, from returning foragers with food, allows the colony to regulate its foraging activity according to the current costs of desiccation and the benefits based on current food availability.

In many harvester ant species, foraging behavior is influenced by the weather [30]–[31]. For example, in the ant *Messor andrei*, recruitment to food bait is higher in more humid conditions [18]. Both humidity and food availability are affected by day to day changes in weather conditions. Food is distributed by wind and flooding and rain uncovers seeds in the top layer of the soil [32]–[33]. In *P. barbatus*, daily changes in conditions such as humidity and food availability produce strong daily trends in the foraging activity of all colonies [24], [34].

Colonies may vary in the relation between humidity and foraging activity. Previous work shows that colonies differ consistently from year to year in how often they forage at all [16]. Most colonies forage on days with high humidity and high food availability, such as those just after a rain when flooding has exposed a layer of seeds in the soil. Few colonies forage on very dry days. Previous work also showed that colonies differ in how likely they are to adjust the rate of outgoing foragers to the rate of forager return [17]. While all colonies tend to adjust outgoing foraging rate closely when conditions are good, only some colonies do so in poor conditions.

Here we asked whether the response of outgoing foragers to returning foragers depends on humidity, and whether the magnitude of response predicts the level of foraging activity. We then considered whether colonies differ in foraging activity, and whether colony differences in foraging behavior persist from year to year.

## Methods

We examined foraging behavior repeatedly in the same colonies over several years and over a range of naturally occurring conditions, so as to encompass the diverse factors that influence colony foraging decisions. To examine how the rate of outgoing foragers depends on the rate of returning foragers, we conducted experiments that manipulated forager return rate. Experiments were performed in August 2009, August-September 2010, and August-September 2011, at the site of a long-term study since 1985 of a population of *P. barbatus* near Rodeo, New Mexico, USA. No permits were required for the field work; permission was granted by Stanford University. Over the course of 3 years we conducted a total of 94 trials with 17 colonies. Three of the 17 colonies were measured in all 3 years, and an additional 2 colonies were measured in 2 of the 3 years. In 2009 there were 32 trials in 8 colonies on 10 days; in 2010 there were 29 trials in 8 colonies on 5 days; in 2011 there were 33 trials in 10 colonies on 12 days (Table S1). All colonies were mature, more than 5 years old (ages determined by yearly census; methods in Gordon and Kulig 1996), except for one colony, 112, which was measured in all 3 years and was 4 years old in 2009.

Returning foragers were prevented from returning to the nest in minutes 4–7 of a 20-min observation period in 2009 and 2010, and a 14-min observation period in 2011. The observation period was reduced because the results from 2009 and 2010 showed that foraging rates did not change much after about 3 minutes following the removal of returning foragers. After the 3 minutes of removals, from minute 8 to the end of the trial, other returning foragers went back to the nest undisturbed, while the ants that had been collected were not released until after the trial. Methods were the same as in [24] and [17]. Rates of returning and outgoing foragers crossing an imaginary line along the trail were measured from video film using an image analysis system developed by Martin Stumpe (http://www.antracks.org). Methods for obtaining the data from image analysis results are described in Prabhakar et al. [35].

As a measure of foraging rate, we used the mean rate of returning foragers per sec. We used the rate of forager return because it reflects both the rate at which foragers went out and food availability. Foraging rate was measured when colonies were undisturbed, before any removals were made. For each trial, the foraging rate was the mean rate of returning foragers per sec from 1 to 240 sec.

To characterize differences among colonies in response to the rate of returning foragers, we used a stochastic version of a threshold model (e.g. [13]). In the model, the probability that foragers leave the nest depends on the rate of forager return. We estimated the colony-specific value of the parameter *c* in a modified version of an algorithm [35] that predicts the flow of outgoing foragers (α*_n_*):
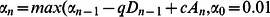
(1)

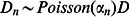
(2)


*A_n_* is the observed number of returning food-bearing foragers at time *n*. To find the value of *c* that best fit the data for each trial, we kept all other parameters fixed at the values that, in previous work, provided a good fit to data from forager removal experiments, predicting the number of outgoing foragers at time *n*, *D_n_*, from the observed rate of returning foragers. The baseline value at which outgoing foragers leave the nest if no foragers return was α_0_ = 0.01, based on previous data from 5 trials of a similar experiment with each of 14 colonies in field experiments in 2008 [17]. If at a given time step very few ants were returning and (α*_n_*_−1_ − *qD_n_*_−1_+ *cA_n_*)<α_0,_ the value of α*_n_* was set to α_0_. The parameter *q*, which represents the reduction in availability of outgoing foragers due to the departure of foragers, was set at 0.05, based on observations inside the nest in field colonies (Pinter-Wollman et al. in review). To estimate *c* for each trial, we used observed rates of returning foragers throughout the trial (*A_n_*), from 0 to 1200 seconds for the 20-min trials from 2009 and 2010, and 0 to 840 sec for the 14-min trials in 2011, and the algorithm from equations 1) and 2) above [35] to simulate the rate of outgoing foragers. For each trial we found c by testing a range of values from 0.01 to 0.25, using the same model-fitting procedures as in [35]: to set a value of *c* for each trial, we compared simulated and observed rates of outgoing foragers in 200 simulations of each value, and chose the value of *c* that gave the lowest root mean square error (RMSE) [36] of the difference between the simulated and observed rates.

We examined whether *c*, the effect of returning foragers on the rate of outgoing foragers, is associated with current humidity. As a measure of humidity we used dew point, the temperature at which water vapor condenses. The higher the humidity, the higher the temperature at which water vapor condenses, and thus dew point is an absolute measure of humidity. Measures of dew point for a given day were obtained from http://www.wunderground.com/showing weather data for San Simon, which tends to have similar conditions as the study area because it is in the same floodplain and at a similar elevation, about 50 km from the study area. We used linear mixed-effects models to test whether foraging rate depends on dew point, and whether the effect of returning foragers on the rate of outgoing foragers (*c*) depends on dew point. Foraging rate and c were the dependent variables in two separate models. In each model, dew point was the main fixed effect and, to account for variation among colonies, we included colony as a random effect. To examine the relation of *c* and foraging rate, we performed a linear regression of *c* on foraging rate, using the mean values of *c* and foraging rate for each colony within each year.

To examine whether colony differences in foraging activity persist from year to year, we first considered the data for 5 colonies for which removal experiments were performed in both 2010 and 2011 (Table S1). We compared the values for 2010 and 2011 for each colony with 2 repeated-measures ANOVAs, either with foraging rate or *c* as dependent variables. Three of these 5 colonies were also tested in 2009. For these 3 colonies we then used 2 different repeated measures ANOVAs with year within colony (2009, 2010, 2011) as main effect and either foraging rate or c as dependent variables.

We then examined variation among colonies in *c* and foraging rate. To test for colony differences, we performed 2 nested ANOVAs with colony, year, and day within year as main effects, and either foraging rate or c as dependent variables.

All statistical tests were conducted using the software R (version 2.12.1).

## Results

**Figure 1:**
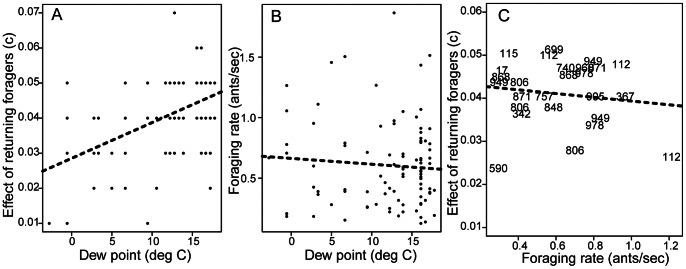
Relation of foraging activity and dew point, a measure of humidity. A. Relation of the effect of returning foragers on the rate of outgoing foragers (*c*) and dew point. B. Relation of foraging rate and dew point. The dotted lines show the least squares fit. C. Relation of effect of returning foragers and forager rate.

The effect of returning foragers on the rate of outgoing foragers, *c*, depends on humidity. The higher the humidity, the higher the value of *c*, or the more each returning forager stimulates other workers to go out and forage. There was a positive association between the effect of returning foragers and dew point (data for all 94 trials of 17 colonies in 3 years; linear mixed-effects model: p<0.0001, Fig. 1A).The increased response to returning foragers when humidity is high is not always sufficient to raise foraging rates. There was no significant relation of foraging rate and dew point (linear mixed-effects model, dew point: p = 0.49, Fig. 1B), and no relation of the effect of returning foragers on outgoing foragers and foraging rate (Fig. 1C) (linear regression: r2 = 0.02, p = 0.48).

**Figure 2:**
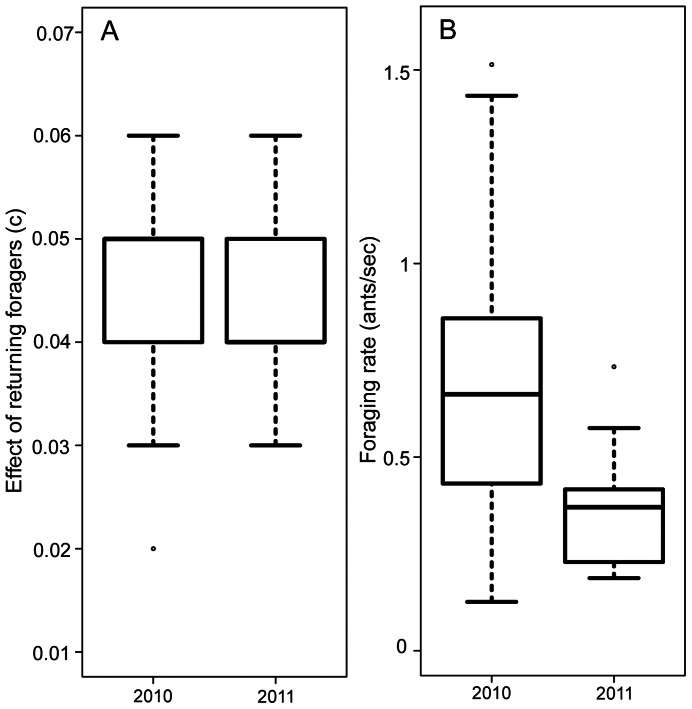
Differences between 2010 and 2011 for 5 colonies. A. The effect of returning foragers on the rate of outgoing foragers (*c*). B. Foraging rate. Boxes indicate the lower and upper quartiles; horizontal lines within boxes indicate the median, whiskers extend to 1.5 interquartile range from the box, and points indicate outliers.

The effect of each returning forager on the rate of outgoing foragers, *c*, appears to shift somewhat from year to year, presumably reflecting changes in conditions. For all 5 colonies tested both in 2010 and 2011, there was no significant difference from 2010 to 2011 in the effect of returning foragers (Repeated measures ANOVA: F = 0.07; df = 1; p = 0.8, Fig. 2A), but over 3 years, for the three colonies that were tested in 2009, 2010, and 2011, there were significant differences among years in the effect of returning foragers (Repeated measures ANOVA: F = 12.4, df = 2, p = 0.02). Foraging rate varied from year to year for the 5 colonies tested in 2010 and 2011 (Repeated measures ANOVA: F = 15.3; df = 1; p = 0.02), and for the three colonies that were tested in 2009, 2010, and 2011 (Repeated measures ANOVA: F = 9.1; df = 2; p = 0.03).

**Figure 3:**
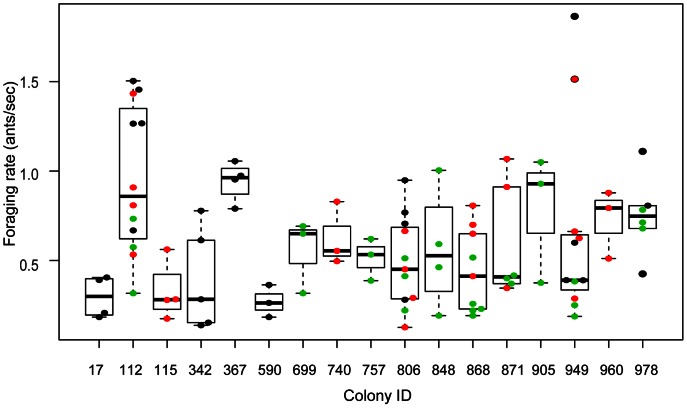
Variation among colonies in foraging rate. Numbers on the x-axis are the colony IDs. Boxes indicate the lower and upper quartiles; horizontal lines within boxes indicate the median, whiskers extend to 1.5 interquartile range from the box. Points represent the values of foraging rate for each trial. Black dots are for trials conducted in 2009, red dots for 2010, and green dots for 2011. Overlapping points are slightly offset along the x axis.

Colonies differed consistently in foraging rate, the rate of returning foragers per second (Fig. 3). There was a significant effect of colony on foraging rate (Table 1), despite significant differences among years (e.g. Fig. 2B) and from day to day. Colonies did not differ in the effect of returning foragers on the rate of outgoing foragers (*c*) (Table 1).

**Table 1:** Results of ANOVAs on effect of returning foragers and foraging rate.

	Overall model			Main effects								
				Colony			Year			Day (nested in year)		
	Adjusted r^2^	F	P	DF	F	P	DF	F	P	DF	F	P
Effect of returning foragers (*c*)	0.39	2.4	**0.001**	16	1.14	0.35	2	13.63	**<0.0001**	24	1.86	**0.03**
Foraging rate (ants/sec)	0.47	2.98	**<0.0001**	16	4.78	**<0.0001**	2	11.98	**<0.0001**	24	1.81	**0.04**

## Discussion

Colonies differ consistently in foraging activity (Fig. 3), and such differences persist from year to year. It is important to note that colony differences in foraging rate do not arise simply from differences in the numbers of ants available to forage. Colony behavior depends on colony size in many ant species (e.g. [37]–[40]). However, differences among colonies are also due to differences in how colonies regulate the activity of their available foragers. For example, some colonies with a low average foraging rate clearly have enough foragers to go out at high rates on some days (e.g. colonies 342 and 949, Fig. 3). Colony variation in foraging rate was also observed in previous work done in a single year [18], and was correlated with variation among colonies in their response to patrollers early in the morning.

Further work is needed to determine why colonies differ in foraging activity. In harvester ants, olfactory cues are used in interactions [25], [41], and there may be variation among colonies in the action of olfactory receptors [42]–[43]. In *Camponotus* ants, workers react to an olfactory stimulus from 5 minutes earlier [44]; such olfactory responses change in the course of an ant’s development [45] and this process may vary among colonies. Finally, differences among colonies in nest structure (e.g. [18], [46]) may influence the strength of chemical cues that ants exchange [47].

Humidity influences the response of outgoing foragers to returning foragers. It is possible that humidity affects the behavior of returning foragers in some way that increases the extent to which returning foragers stimulate those waiting inside the nest entrance. It is also possible that humidity influences the perception of chemical cues in interactions between returning and outgoing forager. Further work is needed to investigate these possibilities. However, humidity does not in itself predict foraging activity. The lack of relation between dew point and foraging rate suggests that within the range of humidity conditions on the days we observed foraging, food availability, as well as any other factors that diminish forager return rate, had a stronger influence on foraging activity than humidity. Though in general, returning foragers tend to stimulate the rate of outgoing foragers more when humidity is higher (Fig. 1A), this does not lead to a significant effect of dew point on foraging rate (Fig. 1C). This suggests that the negative effect of low food availability can override the positive effect of dew point: if food availability is low enough on a humid day, then the rate of forager return will be very low. Even if the effect of each returning forager is high, if the foragers return very infrequently, the rate at which outgoing foragers leave the nest will remain low, which perpetuates a low rate of forager return. Experiments that manipulate food supply in different humidity conditions would help to determine how colony response to food availability and humidity each contribute to foraging rates.

Many factors affect foraging rate at any time, such as temperature [31] and the vegetation ants have to travel through to reach the food [48], while other processes such as predation [49] and amount of stored food [50], probably influence whether a colony forages at all on a given day. Colonies compete with neighbors for foraging area, and colony decisions about interactions with the foragers of neighboring colonies depend on the ages of the colonies involved [32], [51]–[52], so foraging activity also depends on a colony’s age and the age distribution of neighboring colonies. Combining all of these factors, colonies show consistent differences in foraging activity. The question remains open whether colony differences in foraging activity are associated with variation in colony fitness [53].

## Supporting Information

Table S1 **Forager removal experiments by colony and year.** Bold indicates colonies for which trials were performed in all 3 years; italics indicates colonies for which trials were performed in 2 years.
(DOCX)
