# Supplementary material for: Harvester Ant Colony Variation in Foraging Activity and Response to Humidity
Source: PLoS One. 2013 May 23;8(5):e63363. doi: 10.1371/journal.pone.0063363 (PMC3662670; doi:10.1371/journal.pone.0063363)
Supplement: Table S1 — Forager removal experiments by colony and year. Bold indicates colonies for which trials were performed in all 3 years; italics indicates colonies for which trials were performed in 2 years. (DOCX) [file pone.0063363.s001.docx]

Table S1:

Forager removal experiments by colony and year. Bold indicates colonies for which trials were performed in all 3 years; italics indicates colonies for which trials were performed in 2 years.

| Colony | 2009 | 2010 | 2011 |
| --- | --- | --- | --- |
| 17 | X |  |  |
| **112** | **X** | **X** | **X** |
| 342 | X |  |  |
| 367 | X |  |  |
| 590 | X |  |  |
| **806** | **X** | **X** | **X** |
| **949** | **X** | **X** | **X** |
| 978 | X |  | X |
| 115 |  | X |  |
| 740 |  | X |  |
| *868* |  | *X* | *X* |
| *871* |  | *X* | *X* |
| 960 |  | X |  |
| 699 |  |  | X |
| 757 |  |  | X |
| 848 |  |  | X |
| 905 |  |  | X |
